# Antimicrobial stewardship strategies and programs in the outpatient settings: a scoping review

**DOI:** 10.1017/ash.2026.10741

**Published:** 2026-06-11

**Authors:** Adrienne Landsteiner, Muthu Narayan, Jonathan Byrd, Steffi Yang, Kristen Ullman, Catherine Sowerby, Nicholas Zerzan, Caleb Kalinowski, Dimitri Drekonja, Timothy J. Wilt, Wei Duan-Porter

**Affiliations:** 1 CCDOR, Minneapolis VAHCS: Minneapolis VA Medical Center, Minneapolis, USA; 2 Minneapolis VAHCS: Minneapolis VA Medical Center, USA; 3 Medicine, University of Minnesota Medical School, Minneapolis, USA

## Abstract

**Objective:**

To describe the evidence on antimicrobial stewardship programs (ASPs) and strategies implemented in outpatient settings.

**Design:**

We conducted a scoping review to describe the evidence published since 2013 on the effects and implementation of outpatient ASP strategies and programs.

**Methods:**

We searched Ovid MEDLINE, Embase, and Scopus from January 1, 2013 through September 3, 2024. Data were summarized in figures and tables. We categorized studies by types of ASP strategies and settings. We also summarize the reported outcomes using the RE-AIM framework.

**Results:**

Of 29,544 unique citations/abstracts, 285 primary studies and 37 systematic reviews were eligible. Most included primary studies were observational (*k* = 226) and only 59 (21%) were randomized controlled trials (RCT). Most studies were conducted in primary care (*k* = 135) or emergency room/urgent care (*k* = 79). The most commonly evaluated individual strategies were patient and/or clinician education only (*k* = 55), audit and feedback (*k* = 48), and laboratory testing (*k* = 45). Overall antimicrobial and inappropriate prescribing rates were the most frequently assessed outcomes; patient treatment outcomes and health care utilization were also commonly reported.

**Conclusions:**

There has been substantial growth in the evidence base, but this is largely from observational studies. Evidence gaps include a need for more controlled studies, and evaluation of additional outcomes. Few studies evaluated implementation costs or barriers, reach, and sustainability. Many of these additional outcomes would provide valuable insights to aid policy makers in the most feasible and appropriate strategies for implementation.

## Introduction

Antimicrobial stewardship programs (ASPs) seek to improve antimicrobial prescribing practices, optimize patient outcomes, and reduce antimicrobial resistance. More than 80% of antimicrobial usage occurs in the outpatient setting, including primary care clinics, dental clinics, emergency rooms (ER), urgent care, and other sites.^1^ Furthermore, nearly 30% of antibiotics prescribed in the outpatient setting may be inappropriate or unnecessary.^1,2^ Outpatient ASPs have unique challenges, including fewer opportunities to impact prescribing during brief clinical encounters and for one-time prescriptions (compared to inpatient ASPs), the need to tailor strategies for clinics with varying resources and a diversity of clinical populations. In response to these challenges a number of individual strategies and multicomponent ASPs have been developed and implemented in clinical practice.^1^

Here, we present findings from a scoping review that was part of a larger VA Evidence Synthesis Program (ESP) report on the effects and implementation of strategies and multicomponent ASPs in outpatient settings. This review focused on evidence published since 2013, as an update to a previous VA ESP report on the same topic.^3^ The previous review by Drekonja, et al reported most of the available evidence focused on antimicrobial prescribing for respiratory conditions and primarily described changes in prescribing rates but did not report other important clinically meaningful implementation outcomes. We first provide an overall summary of the descriptive information of the included studies and systematic reviews, highlighting the gaps in the evidence as determined by the volume of the evidence base.

## Methods

### Data sources and searches

Ovid MEDLINE, Ovid Embase, and Scopus were searched from January 1, 2013 through September 3, 2024 using key words and subject headings for antimicrobial prophylaxis, drug resistance, antimicrobial stewardship, guideline adherence, evaluation, and specific ASP strategies (*eg*, audit and feedback, clinical decision support system; see Supplement Table 1). Additional citations were identified from hand-searching of systematic review reference lists and consultation with content experts. The protocol was registered a priori on PROSPERO (CRD42024603377).

### Study selection and data abstraction

Abstracts were screened in 2 phases, with the assistance of DistillerSR’s Artificial Intelligence System (DAISY) machine learning tool in the screening process.^4^ In the first phase, 10,441 abstracts were screened and exclusion of abstracts required consensus by 2 human reviewers. In phase 2, all remaining abstracts had a predicted likelihood of inclusion ≤ 0.1 and eligibility was determined by a single human reviewer; 6,368 abstracts were screened during this phase and none were found to be eligible (Supplement Table 2). Finally, the remaining 12,735 abstracts were not examined by a human reviewer. For full-text review, 2 investigators independently reviewed, and eligibility required consensus, when consensus could not be reached discussion with third individual was required.

From all eligible studies, study design, population (adults), setting, intervention, and reported outcomes were abstracted. Data abstraction was completed by 1 reviewer and verified by a second.

### Data synthesis and analysis

We summarized study characteristics for eligible studies and systematic reviews using descriptive figures and tables. We categorized primary studies by types of ASPs and strategies, and for systematic reviews, we identified the types of strategies included by these reviews. ASPs and strategies were categorized by the primary method of behavioral change, such as audit and feedback or a clinical decision support system. We used author descriptions of interventions to identify and group the ASP strategy or program. When multiple methods were present in an ASP those studies were categorized as multicomponent. We also identify and report all the eligible outcomes reported by studies and systematic reviews. We summarize these outcomes with the use of the RE-AIM framework (Supplement Table 3).

### Quality and certainty of evidence

As this was a scoping review with the intention of mapping the existing research on the broad topic of investigating implementation of ASPs, we did not conduct risk of bias assessments or rate the certainty of the evidence.

## Results

**Figure 1.**
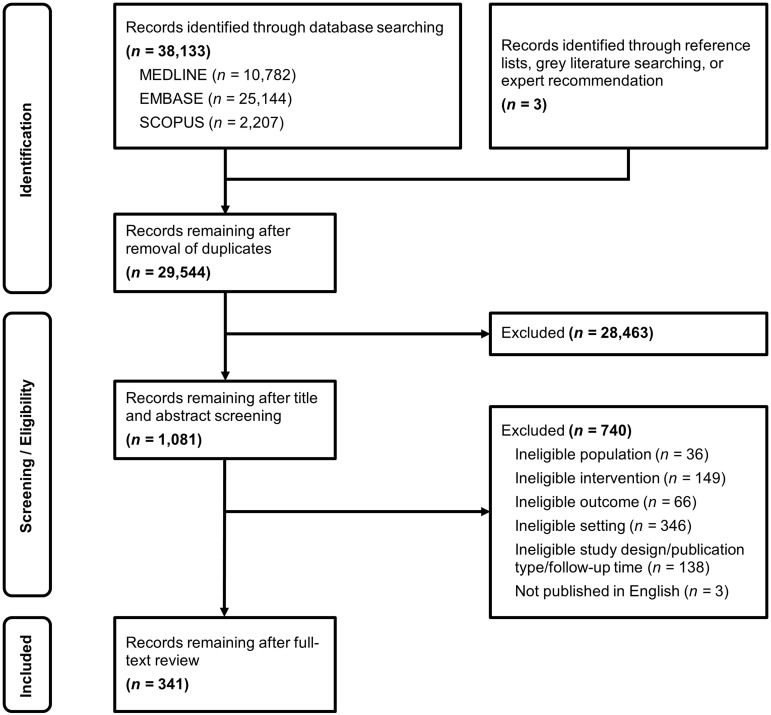
Figure 1 long description. Literature flow diagram.

From 29,544 unique citations, we identified 285 unique primary studies (reported in 322 articles) and 37 systematic reviews, for a total of 341 included studies (Figure 1). Most of studies were observational (*k* = 226), including 41 with contemporaneous comparators; only a fifth of studies (*k* = 59) were RCTs. A quarter of primary studies were conducted at a single site (*k* = 69), a fifth included 2–10 sites (*k* = 53), and less than a tenth included more than 100 sites (*k* = 25). The studies with more than 100 sites took place in either large health systems and/or across large geographic areas (*eg*, involving regional or national health systems or networks).

### Overview of included primary studies

Most of primary studies were conducted in primary care (*k* = 135) or ER/urgent care settings (*k* = 79). A small number were conducted in non-specific outpatient settings (*k* = 32) or across multiple settings (*k* = 18), with all those in the latter group including sites in primary care and/or ER/urgent care. Few studies were conducted in outpatient pharmacy (*k* = 10), outpatient surgery (*k* = 4), dental clinics (*k* = 6), or outpatient dialysis (*k* = 1).

**Figure 2.**
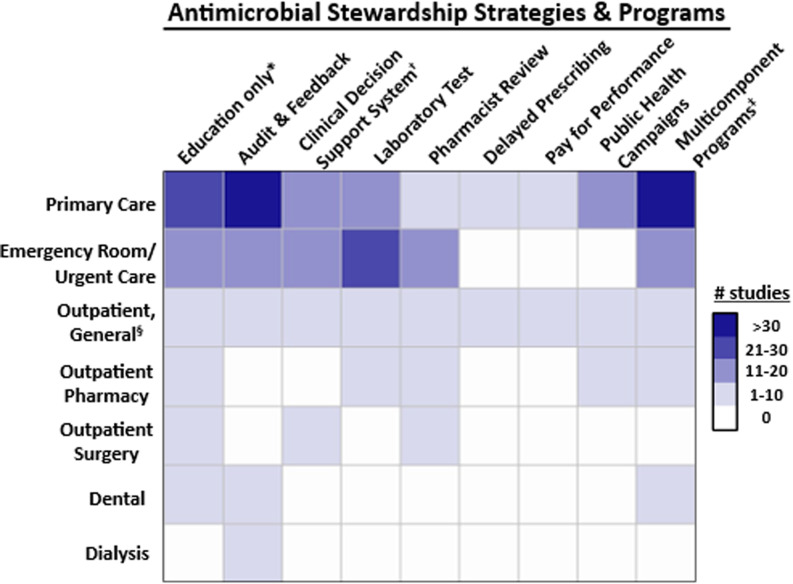
Figure 2 long description. Antimicrobial stewardship strategies across various outpatient settings. *Notes*: *Patient and/or clinician education. ^†^Tools embedded in electronic health records with computerized order entry. ^‡^Programs included 2 or more specific strategies, in addition to patient and/or clinician education. ^§^Non-specific outpatient setting or multiple types of outpatient clinics (*eg*, primary care and medical specialties).

The most commonly evaluated strategies were patient and/or clinician education only (*k* = 55), audit and feedback (*k* = 48), and laboratory testing (*k* = 45). Multicomponent ASPs were also frequently examined and reported in 56 studies. Fewer studies specifically focused on clinical decision support systems (CDSS) (*k* = 35), public health campaigns (*k* = 22), pharmacist review (*k* = 15), delayed prescribing (*k* = 7), or pay for performance programs (*k* = 2). Similar patterns were seen for frequency of ASPs or strategies being examined across different outpatient settings (Figure 2).

Below we summarize the evidence by setting (primary care, emergency room/urgent care, and other outpatient settings), followed by a summary of the included systematic reviews.

#### Primary care setting

**Figure 3.**
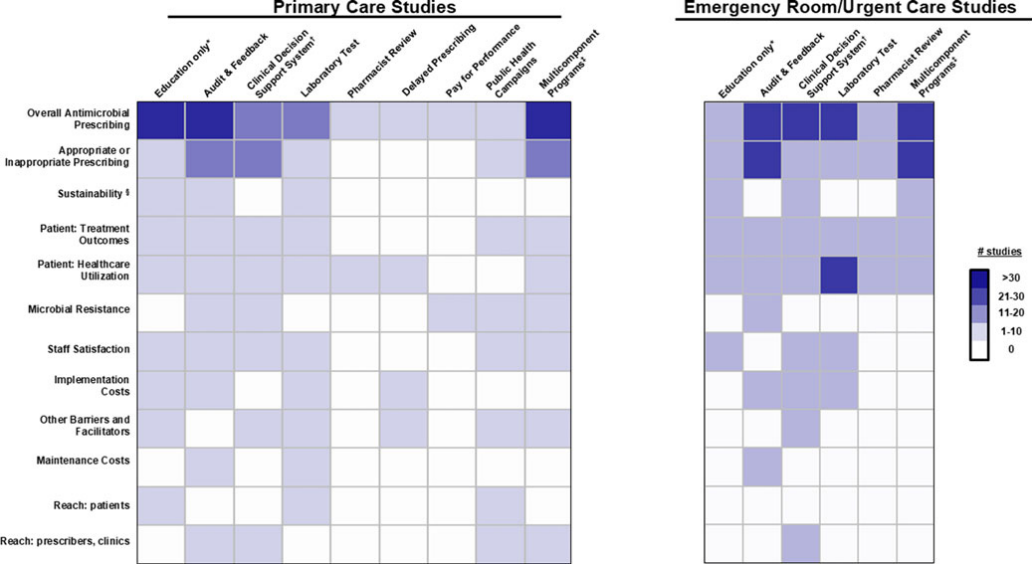
Figure 3 long description. Primary care: Strategies used in multicomponent antimicrobial stewardship programs. *Abbreviations.* A/F, audit and feedback; CDSS, clinical decision support system; Delay Rx, delayed prescribing; Pub health, public health campaign; P4P, pay for performance.

Most of the included studies set in primary care were observational (*k* = 103) in design with only a fifth of these having concurrent comparators (*k* = 21). Forty-eight studies were RCTs. Table 1 summarizes the characteristics of primary care studies categorized by specific ASPs. The most frequently implemented strategies were audit and feedback (*k* = 35) and patient/clinician education alone (*k* = 27). Multicomponent ASPs were also commonly evaluated (*k* = 32). For studies evaluating multicomponent ASPs, the most common combination was audit and feedback paired with CDSS (*k* = 17); with a few studies incorporating a third component such as delayed prescribing or pay for performance (Figure 3). The remaining studies evaluated audit and feedback or CDSS combined with other strategies (*k* = 9), public health campaigns with pay for performance (*k* = 4), or laboratory test with either delayed prescribing or formulary restrictions (*k* = 2).

**Table 1.** Summary characteristics of primary care studies Table 1 long description.

Characteristic	Characteristic grouping	Education only* *(k = 27)*	Audit and feedback *(k = 35)*	CDSS^†^ *(k = 17)*	Lab tests *(k = 20)*	Public health *(k = 13)*	Pharmacist review *(k = 1)*	Delayed prescribing *(k = 5)*	Pay for performance *(k = 1)*	Multi- component^‡^ *(k = 32)*
**Study design**	RCT	10	15	3	8	─	─	1	─	11
	Observational concurrent comparator	6	6	─	4	1	─	─	─	4
	Observational single group	11	14	14	8	12	1	4	1	17
**# Prescribers**	<50	7	6	3	9	─	1	─	─	2
	50–200	6	8	3	4	─	─	─	─	4
	201–500	2	1	─	4	2	─	─	─	4
	501–1,000	2	1	2	─	1	─	─	─	1
	>1,000	2	6	─	─	3	─	1	─	─
**# Clinic sites**	1	5	3	8	1	─	1	─	─	3
	2–10	3	8	3	4	2	─	1	─	4
	11–50	3	4	2	3	1	─	─	─	8
	51–100	1	3	1	3	1	─	1	─	2
	101–200	2	1	2	─	1	─	─	─	3
	>200	─	4	─	2	2	─	─	1	2
	Very large national studies^§^	─	─	─	─	─	─	1	─	─
**Outcomes**	Overall antimicrobial prescribing	23	30	16	14	7	1	3	1	28
	Inappropriate prescribing	5	16	11	4	2	─	─	─	15
	Sustainability in prescribing^¶^	2	1	─	1	─	─	─	─	─
	Patient: treatment outcomes	6	7	4	9	2	─	─	─	5
	Patient: health care utilization**	1	8	5	5	─	1	1	─	4
	Microbial resistance	─	1	1	─	2	─	─	1	1
	Staff satisfaction	5	4	1	6	2	─	─	─	3
	Implementation costs	1	4	─	3	─	─	1	─	─
	Other barriers and facilitators	1	─	1	7	1	─	1	─	4
	Maintenance costs	─	2	─	1	─	─	─	─	─
	Reach: patients, community	1	─	─	1	2	─	─	─	1
	Reach: prescribers, facilities	─	2	2	─	2	─	─	─	2

Notes. *Patient and/or clinician education.

^†^Tools embedded in electronic health records with computerized order entry.

^‡^Programs included 2 or more specific strategies, in addition to patient and/or clinician education.

^§^Includes 1 study in England and Wales with a patient population of 1.8 million.^7^

^¶^Long-term effects on antimicrobial prescribing, at least 1 year after (and in a separate phase) from initial implementation.

**Includes hospital admissions, emergency room, and office visits.

*Abbreviations.* CDSS, clinical decision support system; RCT, randomized controlled trial.

Table 1 summarizes the most commonly reported outcomes in the studies evaluating ASPs in primary care settings. The most commonly reported outcomes were overall antimicrobial use (*k* = 123, 82%), followed by inappropriate or appropriate antimicrobial prescribing (*k* = 53, 34%). Less than a quarter of studies assessed patient treatment outcomes (*k* = 33) or health care utilization (*k* = 25), and fewer examined staff satisfaction (*k* = 21). Rarely assessed outcomes include implementation barriers and facilitators (*k* = 15) or costs (*k* = 9), reach among patients (*k* = 5) or prescribers (*k* = 8), and sustainability (*k* = 4) or maintenance costs (*k* = 3).

#### Emergency room/urgent care setting

Ninety-five studies examined ASPs implemented in an ER or urgent care setting, of which 87 were observational in design and 8 were RCTs. Table 2 summarizes characteristics for studies conducted in ER/urgent care by specific antimicrobial strategy or multicomponent ASP. The most commonly used strategy was laboratory test (*k* = 21), followed by multicomponent ASPs (*k* = 18). Other individual strategies were patient/clinician education alone (*k* = 15), CDSS (*k* = 15), audit and feedback (*k* = 15), and pharmacist review (*k* = 11). No studies evaluated delayed prescribing, pay for performance measures, or public health campaigns.

**Table 2.** Summary characteristics of primary studies conducted in emergency room/Urgent care Table 2 long description.

Characteristic	Characteristic grouping	Education only* *(k = 15)*	Audit and feedback *(k = 15)*	CDSS^†^ *(k = 15)*	Lab tests *(k = 21)*	Pharmacist review *(k = 11)*	Multi-component^‡^ *(k = 18)*
**Study design**	RCT	1	─	─	7	─	─
	Observational concurrent comparator	1	3	3	6	─	1
	Observational single group	13	12	12	8	11	17
**# Prescribers**	<50	3	1	2	1	─	1
	50–200	2	3	1	─	─	2
	201–500	─	─	─	─	─	1
	501–1,000	─	─	1	─	─	─
	>1,000	─	─	1	─	─	─
**# Clinic sites**	1	8	7	4	8	6	7
	2–10	4	5	4	8	2	4
	11–50	─	1	2	1	─	3
	101–200	─	─	2	─	─	─
**Outcomes**	Overall antimicrobial prescribing	10	12	11	18	10	15
	Inappropriate prescribing	7	12	8	4	6	11
	Sustainability in prescribing^¶^	1	─	1	─	─	1
	Patient: treatment outcomes	4	6	5	7	2	2
	Patient: health care utilization^§^	4	6	5	12	6	9
	Microbial resistance	─	1	─	─	─	─
	Staff satisfaction	2	─	2	2	─	─
	Implementation costs	─	1	1	1	─	─
	Other barriers and facilitators	─	─	1	─	─	─
	Maintenance costs	─	1	─	─	─	─
	Reach: prescribers, facilities	─	─	1	─	─	─

Notes. *Patient and/or clinician education.

^†^Tools embedded in electronic health records with computerized order entry.

^‡^Programs included 2 or more specific strategies, in addition to patient and/or clinician education.

^§^Includes hospital admissions, emergency room, and office visits.

For studies evaluating multicomponent ASPs in ER/urgent care settings, the most common combination was audit and feedback with CDSS (*k* = 9; Figure 3). The remaining studies examined audit and feedback or CDSS combined with other strategies (*k* = 7), or laboratory tests with pharmacist review (*k* = 2).

Similar to studies conducted in a primary care setting, change in overall antimicrobial prescribing was the most frequently reported outcome (*k* = 76, 80%). Inappropriate or appropriate prescribing (*k* = 42, 47%) were also frequently reported. Patient treatment outcomes were reported in 27 studies, staff satisfaction in 6, sustainability of prescribing changes in 3, and implementation costs were reported in 3 studies. Change in antimicrobial resistance patterns, implementation barriers and facilitators, maintenance costs, and reach among healthcare professionals was rarely reported, with only a single study reporting a measure of reach. No study assessed reach among patients or communities. Outcome measurement was similar across studies examining specific strategies. Changes in overall antimicrobial prescribing is the most commonly reported outcome regardless of strategy.

#### Other outpatient settings

The remaining 58 studies were conducted in a variety of settings, including general non-specific outpatient (or multiple clinic types; *k* = 36), outpatient pharmacy (*k* = 11), dental clinic (*k* = 6), outpatient surgery (*k* = 4), and dialysis facilities (*k* = 1). Fifty-four of these studies were observational studies, of which 7 had concurrent comparators. Four were RCTs. Table 3 summarizes the characteristics of these studies, categorized by antimicrobial stewardship strategy or program. These studies most often evaluated patient/clinician education alone (*k* = 13), CDSS (*k* = 11), multicomponent ASPs (*k* = 10), or public health campaigns (*k* = 9). Fewer studies examined audit and feedback (*k* = 4), laboratory tests (*k* = 4), pharmacist review (*k* = 3), and delayed prescribing (*k* = 3). Only 1 study evaluated pay for performance. Most of the multicomponent ASPs included audit and feedback with 1 or more additional strategy (*k* = 7).

**Table 3.** Summary characteristics of studies conducted in other outpatient settings Table 3 long description.

Characteristic	Characteristic grouping	Education only* *(k = 13)*	Audit and feedback *(k = 4)*	CDSS^†^ *(k = 11)*	Lab tests *(k = 4)*	Public health *(k = 9)*	Pharmacist review *(k = 3)*	Delayed prescribing *(k = 3)*	Pay for performance *(k = 1)*	Multi- component^‡^ *(k = 10)*
**Setting**	Outpatient, general	6	1	10	2	6	1	3	1	6
	Outpatient surgery	2	─	1	─	─	1	─	─	─
	Outpatient pharmacy	2	─	─	2	3	1	─	─	3
	Dialysis	─	1	─	─	─	─	─	─	─
	Dental	3	2	─	─	─	─	─	─	1
**Study design**	RCT	1	─	─	─	─	─	2	─	1
	Observational concurrent comparator	3	─	1	1	2	─	─	─	─
	Observational single group	9	4	10	3	7	3	1	1	9
**# Prescribers**	<50	1	3	─	1	─	─	─	─	─
	50–200	1	─	─	─	─	─	1	─	1
	>1,000	─	─	1	─	─	─	─	─	─
**# Clinic sites**	1	4	─	3	─	1	1	─	─	2
	2–10	1	1	─	─	─	─	─	─	1
	11–50	─	─	1	─	─	─	2	─	─
	51–100	─	─	─	1	─	─	─	─	1
	>200	2	─	─	─	─	1	─	─	─
	**Very large national studies** ^§^	─	─	─	─	─	1	1	1	─
**Outcomes** ^¶^	Overall antimicrobial prescribing	10	3	9	3	9	2	2	1	9
	Inappropriate prescribing	2	3	6	1	3	─	─	─	3
	Patient: treatment outcomes	4	1	2	1	2	1	1	─	3
	Patient: health care utilization**	3	1	4	2	─	─	1	─	2
	Microbial resistance	1	─	1	─	─	1	─	─	1
	Staff satisfaction	1	─	─	1	─	1	1	─	2
	Implementation costs	─	─	1	1	1	─	1	─	3
	Other barriers and facilitators	─	1	─	1	─	1	1	─	1
	Maintenance costs	─	─	─	─	1	─	─	─	1
	Reach: prescribers, facilities	─	─	─	─	─	1	─	─	─

Notes. *Patient and/or clinician education.

^†^Tools embedded in electronic health records with computerized order entry.

^‡^Programs included 2 or more specific strategies, in addition to patient and/or clinician education.

^§^Two studies in Japan included a population of 2.9 million patients^8^ and 13.5 million patients.^9^ One study in England and Wales included a population of 1.8 million patients.^7^

^¶^No studies reported sustainability in effects on prescribing or reach for patients and communities.

**Includes hospital admissions, emergency room, and office visits.

*Abbreviations*. CDSS, clinical decision support system; RCT, randomized controlled trial.

The most frequently reported outcome was change in overall antimicrobial prescribing (*k* = 48, 83%), followed by inappropriate or appropriate prescribing (*k* = 18, 31%), patient treatment outcomes (*k* = 15, 26%), or health care utilization (*k* = 13, 22%). All other outcomes were rarely reported and no study examined sustainability of prescribing changes or reach among patients. These patterns of outcome reporting were similar across studies, regardless of the evaluated specific strategy or program (Table 3).

### Included systematic reviews

Thirty-seven systematic reviews were eligible and each review included between 10–59 studies. Reviews addressed various outpatient settings, including primary care (*k* = 17), ER/urgent care (*k* = 5), general outpatient *(k* = 5), outpatient pharmacy (*k* = 2), and dental clinics (*k* = 1). Seven of the reviews included multiple outpatient settings. Reviews included a number of stewardship strategies or programs including education only, audit and feedback, CDSS, and laboratory tests being the most common. The most frequently reported outcomes were changes in antimicrobial prescribing (*k* = 34), patient treatment outcomes (*k* = 20), and health care utilization (*k* = 12). Ten reviews examined inappropriate or appropriate prescribing, four addressed implementation costs, three reported barriers and facilitators, two reported on staff satisfaction, a single review reported on changes in microbial resistance, and a single review reported on maintenance costs.

## Discussion

The number of primary studies evaluating ASP and strategies has grown substantially since 2013, but most of this evidence is observational studies without any concurrent comparators. Additionally, the evidence base is dominated by studies evaluating only education or audit and feedback. Change in overall antimicrobial prescribing and inappropriate (or appropriate) prescribing rates were the outcomes most frequently reported. This is unsurprising since reduction of antimicrobial usage is the main goal of most ASPs and strategies. In contrast, whereas avoiding community antibiotic resistance is an important long-term goal of ASPs, this is much more challenging to assess and thus was rarely reported by studies. While patient treatment outcomes and health care utilization were also commonly reported, very few studies evaluated staff satisfaction and acceptability, implementation costs and barriers, sustainability, or reach.

Understanding implementation outcomes such as staff acceptability, sustainability, and reach, would allow for greater exploration of why and how an ASP was effective or ineffective, leading to further optimization or possible reframing and execution. A priori planning to assess implementation metrics will ensure these outcomes are captured comprehensively and in a valid and reproducible manner. Our findings suggest that future research should consider outcomes beyond overall antimicrobial prescribing or inappropriate prescribing to improve our understanding of why ASPs may be effective and to enhance future implementation efforts. In the United States, medical specialties that contribute substantially to outpatient antibiotic prescribing (outside of primary care) include various surgical specialties, dentistry, emergency medicine, dermatology, and obstetrics/gynecology.^5^ Most of the identified studies in this review were conducted in either primary care or emergency room settings, considering that published estimates of dental practice prescribing is 10% of all outpatient prescriptions and an estimated 80% of those may be unnecessary, this setting is under represented in the evidence base.^6^ Research into these other outpatient settings is necessary to ascertain the degree to which prescribing may be inappropriate and if so what approach may be the most effective in addressing antimicrobial stewardship.

This review had limitations. We limited our search to English-language studies and reviews and as such, we may have missed relevant publications in non-English languages. However, included studies were conducted in varying healthcare systems and outpatient settings in many different countries. Additionally, we used AI tools to assist with abstract screening, and 40% of abstracts were not additionally reviewed by a human reviewer. The estimated impact of using DistillerSR DAISY during the abstract screening process is minimal. Using the DistillerSR predictive estimates, the number of eligible articles that may have been missed was 4, which would equal 1% of the identified eligible articles. It is unlikely that this smaller number of articles would have substantially impacted the overall characteristics of the evidence base.

## Conclusions

There has been substantial growth in the evidence base, but this is largely from observational studies. Evidence gaps include a need for more controlled studies, and evaluation of additional outcomes, including implementation cost or barriers, reach, and sustainability. Many of these additional outcomes would provide valuable insights to aid policy makers in the most feasible and appropriate strategies to implement in their health care systems.

## Supporting information

10.1017/ash.2026.10741.sm001 Landsteiner et al. supplementary material Landsteiner et al. supplementary material

## Supplementary material

The supplementary material for this article can be found at https://doi.org/10.1017/ash.2026.10741.

## Figure 1. Long description

The flowchart begins with the identification phase, where records are identified through database searching in MEDLINE, EMBASE, and SCOPUS, totaling 38,133 records. Additionally, 3 records are identified through reference lists, grey literature searching, or expert recommendation. After removing duplicates, 29,544 records remain. In the screening and eligibility phase, 28,463 records are excluded, leaving 1,081 records after title and abstract screening. Further exclusions are made for ineligibility in population, intervention, outcome, setting, study design, publication type, follow-up time, and non-English publications, totaling 740 exclusions. Finally, 341 records remain after full-text review.

Navigate back to Figure 1..

## Figure 2. Long description

A heat map displays antimicrobial stewardship strategies across various outpatient settings. The heat map is divided into rows representing different outpatient settings such as Primary Care, Emergency Room/Urgent Care, Outpatient General, Outpatient Pharmacy, Outpatient Surgery, Dental, and Dialysis. The columns represent different strategies including Education Only, Audit & Feedback, Clinical Decision Support System, Laboratory Test, Pharmacist Review, Delayed Prescribing, Pay for Performance, Public Health Campaigns, and Multicomponent Programs. The color intensity indicates the number of studies, with darker shades representing a higher number of studies. Primary Care shows the highest concentration of studies across multiple strategies, particularly in Education Only, Audit & Feedback, and Multicomponent Programs. Emergency Room/Urgent Care also shows significant activity in several strategies. Other settings like Outpatient Pharmacy, Outpatient Surgery, Dental, and Dialysis have fewer studies overall.

Navigate back to Figure 2..

## Figure 3. Long description

The matrix compares strategies used in multicomponent antimicrobial stewardship programs across primary care and emergency room settings. It features two sections: Primary Care Studies and Emergency Room/Urgent Care Studies. Each section is organized into rows representing different outcomes such as overall antimicrobial prescribing, appropriate or inappropriate prescribing, sustainability, patient treatment outcomes, healthcare utilization, microbial resistance, staff satisfaction, implementation costs, other barriers and facilitators, maintenance costs, and reach for patients and prescribers. The columns represent various strategies including education only, audit and feedback, clinical decision support system, laboratory test, pharmacist review, delayed prescribing, pay for performance, public health campaigns, and multicomponent programs. The color intensity indicates the number of studies, with darker shades representing a higher number of studies. Notable trends include a higher concentration of studies on overall antimicrobial prescribing and appropriate or inappropriate prescribing in both primary care and emergency room settings.

Navigate back to Figure 3..

## Table 1. Long description

A table comparing characteristics of primary care studies by intervention type and study design. The table has 13 rows and 10 columns. The columns are labeled with characteristic groupings such as Education only, Audit and feedback, CDSS, Lab tests, Public health, Pharmacist review, Delayed prescribing, Pay for performance, and Multi-component. The rows are labeled with study design, number of prescribers, number of clinic sites, and outcomes. Each cell contains the number of studies corresponding to each characteristic grouping. Notable trends include a higher number of studies using audit and feedback and education only, as well as a significant number of multi-component studies.

Navigate back to Table 1..

## Table 2. Long description

A table comparing characteristics of primary studies conducted in emergency room and urgent care settings by specific antimicrobial strategy or multicomponent antimicrobial stewardship programs. The table has 18 rows and 7 columns. The columns are labeled as Characteristic, Characteristic grouping, Education only, Audit and feedback, CDSS, Lab tests, Pharmacist review, and Multi-component. The rows are labeled as Study design, Number of Prescribers, Number of Clinic sites, and Outcomes. Each cell contains numerical values representing the number of studies for each characteristic and strategy. Notable trends include the most commonly used strategy being laboratory tests followed by multicomponent antimicrobial stewardship programs. Other strategies include patient and clinician education alone, clinical decision support systems, audit and feedback, and pharmacist review.

Navigate back to Table 2..

## Table 3. Long description

A table summarizing characteristics of studies conducted in various outpatient settings, categorized by antimicrobial stewardship strategies. The table includes 11 columns representing different strategies such as education only, audit and feedback, CDSS, lab tests, public health, pharmacist review, delayed prescribing, pay for performance, and multicomponent. Each strategy is further broken down by setting, study design, number of prescribers, number of clinic sites, and outcomes. The settings include outpatient general, outpatient surgery, outpatient pharmacy, dialysis, and dental. Study designs include RCT and observational studies with or without concurrent comparators. The number of prescribers and clinic sites varies across studies. Outcomes measured include overall antimicrobial prescribing, inappropriate prescribing, patient treatment outcomes, patient health care utilization, microbial resistance, staff satisfaction, implementation costs, other barriers and facilitators, maintenance costs, and reach. The table provides a detailed comparison of the frequency of each characteristic across different strategies.

Navigate back to Table 3..
