# Supplementary material for: Antimicrobial stewardship strategies and programs in the outpatient settings: a scoping review
Source: Antimicrob Steward Healthc Epidemiol. 2026 Jun 11;6(1):e176. doi: 10.1017/ash.2026.10741 (PMC13273154; doi:10.1017/ash.2026.10741)
Supplement: Landsteiner et al. supplementary material [file S2732494X26107414sup001.docx]

Supplement

Table 1. Medline Search Strategy

| **#** | **Search Statement** | **Results** |
| --- | --- | --- |
|  | Antibiotic Prophylaxis/ or exp anti-bacterial agents/ or anti-infective agents, urinary/ | 855085 |
|  | (antibacteri* or anti-bacteri* or antibiotic* or antimicrob* or anti-microb* or (anti-infective* adj3 urinary)).ti,ab,kf. | 706676 |
|  | exp Aminoglycosides/ or exp Carbapenems/ or exp Carbapenem-Resistant Enterobacteriaceae/ or exp Cephalosporins/ or exp Fluoroquinolones/ or Methicillin-Resistant Staphylococcus aureus/ or Vancomycin/ or Vancomycin-Resistant Staphylococcus aureus/ or Vancomycin-Resistant Enterococci/ | 294357 |
|  | (aminoglycoside? or carbapenem? or cephalosporin? or fluoroquinolone? or vancomycin?).ti,ab,kf. | 111077 |
|  | exp Drug Resistance, Bacterial/ or drug resistan* bacteria?.ti,ab,kf. or beta-lactam* resistan*.ti,ab,kf. | 111381 |
|  | or/1-5 | 1337123 |
|  | Antimicrobial Stewardship/ or ((antibacteri* or anti-bacteri* or anti-infecti* or antimicrobial? or anti-microbial? or bacteria* or aminoglycoside? or beta-lactam* or broad spectrum* or carbapenem? or cephalosporin? or fluoroquinolone? or vancomycin?) adj3 steward*).ti,ab,kf. | 9852 |
|  | (Education/ or exp Curriculum/ or exp Health Education/ or exp Education, Professional/ or Formative Feedback/ or Interdisciplinary Placement/ or exp Inservice Training/ or Interprofessional Education/ or Staff Development/ or exp Professional Competence/ or ed.fs.) not Education, Veterinary/ | 841356 |
|  | ((clinic* or nurse? or nursing or pharmac* or physician or practitioner? or professional or provider?) adj3 (educat* or feedback or inservice? or outreach or perspective? or teach* or train*)).ti,ab,kf. | 196772 |
|  | decision making, computer-assisted/ or decision support systems, clinical/ or exp electronic health records/ or drug therapy, computer-assisted/ or Electronic Prescribing/ or medical order entry systems/ or medical records systems, computerized/ or Point-of-Care Systems/ or Point-of-Care Testing/ or reminder systems/ or ((computer* or electronic* or EHR or internet or online or system? or web-based) adj3 (assist* or decision* or nudge? or order? or prescrib* or prescrip* or prompt? or reminder? or record? or support or tool?)).ti,ab,kf. or (point adj2 care).ti,ab,kf. | 280202 |
|  | exp Clinical Decision-Making/ or Decision-Making/ or ((clinic* adj2 support*) or "decision making" or "decision support*" or "expert* system?").ti,ab,kf. | 345276 |
|  | Drug Prescriptions/ or ((direction? or directive?) adj3 (prescrib* or prescrip*)).ti,ab,kf. | 31552 |
|  | Drug Utilization Review/ or drug utili?ation review?.ti,ab,kf. or drug utili?ation evaluation.ti,ab,kf. | 4338 |
|  | exp Clinical Audit/ or Management Audit/ or (audit or audits or auditing).ti,ab,kf. | 62630 |
|  | Guideline Adherence/ or Guidelines as Topic/ or Practice Guidelines as Topic/ or ((guideline? or pathway? or protocol? or recommend? or requirement? or standard*) adj3 (adherence or concordance or implement* or practice?)).ti,ab,kf. | 280108 |
|  | Inappropriate Prescribing/ or ((appropriate* or decrease* or de-escalat* or inappropriate* or unnecessary) adj3 prescrib*).ti,ab,kf. | 10908 |
|  | Consumer Health Information/ or Health Communication/ or Information Dissemination/ or exp "Marketing of Health Services"/ or Patient Education as Topic/ or Patient Medication Knowledge/ or ((consumer? or patient? or public) adj3 (awareness or communication or educat* or informational* or literacy or perspective? or teach*)).ti,ab,kf. or (campaign* or marketing).ti,ab,kf. or (information* adj2 disseminat*).ti,ab,kf. or (patient* adj3 knowledg*).ti,ab,kf. | 364695 |
|  | interdisciplinary communication/ or Interprofessional Relations/ or Leadership/ or ((inter-disciplin* or interdisciplin* or inter-profession* or interprofession* or multidisciplin* or transdisciplin*) adj3 (communicat* or leadership or management or relation*)).ti,ab,kf. | 129782 |
|  | Medication Review/ or exp medication systems/ or (medication adj3 (reconciliation or review* or system?)).ti,ab,kf. | 13486 |
|  | Patient Discharge/ or ((hospital* or patient?) adj2 discharg*).ti,ab,kf. | 120857 |
|  | Physician Incentive Plans/ or Prior Authorization/ or Reimbursement Mechanisms/ or ((physician or reimburse*) adj3 incent*).ti,ab,kf. or (preauthorization or pre-authorization or prior authorization?).ti,ab,kf. | 17402 |
|  | Practice Patterns, Nurses'/ or Practice Patterns, Pharmacists'/ or Physician's Practice Patterns/ or (prescrib* adj3 practice?).ti,ab,kf. | 76351 |
|  | Program Evaluation/ or Quality Improvement/ or (quality adj3 improve*).ti,ab,kf. or ((intervention* or program*) adj3 (effectiveness or evaluat* or implement*)).ti,ab,kf. | 409232 |
|  | or/7-23 | 2509285 |
|  | and/6,24 | 64354 |
|  | 25 not (exp animals/ not humans.sh.) | 62719 |
|  | 26 not (exp infant/ or exp child/ or adolescent/) not exp adult/ | 36972 |
|  | 27 not (animal or bovine or canine or cattle or chicken? or cow or cows or farm or farms or horse? or livestock or mice or mouse or murine or pig or pigs or piglet? or porcine or poultry or rabbit? or sheep or swine or veterinary or veterinarian? or child or children or infant? or neonatal or p?ediatric*).jn,ti. | 35262 |
|  | 28 not (case reports/ or comment/ or editorial/ or letter/ or news/ or study guide/) | 31638 |
|  | limit 29 to (english language and yr="2013 -Current") | 19964 |
|  | exp randomized controlled trial/ | 622463 |
|  | controlled clinical trial/ | 95599 |
|  | exp clinical trial/ | 1003355 |
|  | (clin$ adj3 trial$).ti,ab,kw. | 564769 |
|  | Drug Therapy.fs. | 2730915 |
|  | groups.ab. | 2729886 |
|  | controlled trial.ti,ab,kw. | 189281 |
|  | ((singl$ or doubl$ or trebl$ or tripl$) adj25 (blind$ or mask$)).ti,ab,kw. | 218499 |
|  | Placebos/ | 35994 |
|  | placebo$.ti,ab,kw. | 260614 |
|  | random$.ti,ab,kw. | 1553468 |
|  | (Observational Study or Clinical Study).pt. | 167140 |
|  | (observational adj3 (study or studies or design or analysis or analyses)).ti,ab,kf. | 252938 |
|  | (cohort* adj3 (study or studies or design or analysis or analyses)).ti,ab,kf. | 444939 |
|  | (prospective adj7 (study or studies or design or analysis or analyses)).ti,ab,kf. | 576869 |
|  | ((follow up or followup) adj7 (study or studies or design or analysis or analyses)).ti,ab,kf. | 182920 |
|  | ((longitudinal or longterm or (long adj term)) adj7 (study or studies or design or analysis or analyses or data)).ti,ab,kf. | 379796 |
|  | (quasi adj (experiment or experiments or experimental)).ti,ab,kf. | 23379 |
|  | organizational case studies/ or ((institution* or organization*) adj3 (evaluation or study)).ti,ab,kf. | 36291 |
|  | Interrupted Time Series Analysis/ or ((segment$2 adj3 regression) or time* series or slope change or ((piecewise or piece-wise) adj3 regression)).tw,kf. or ((implement* or rates) and (((pre or before or prior) adj5 (post or after or follow*)) or quasi-experiment* or quasiexperiment* or natural experiment* or ARIMA or autoregress* or auto-regress* or integrat* moving average or segmented or segments or piecewise or piece-wise)).tw,kf. | 155488 |
|  | exp Empirical Research/ or Interviews as Topic/ or Focus Groups/ or Narrative Medicine/ or Research Design/ or "Surveys and Questionnaires"/ or (focus group? or hybrid design or interview* or grounded theory or mixed method? or purposiv* sampling or qualitativ* or survey* or theme?).ti,ab. or ((concept* or data or thematic or theoretical) adj2 saturat*).ti,ab. | 2117965 |
|  | or/31-51 | 9036353 |
|  | 30 and 52 | 10782 |
|  | ***Total*** | **10,782** |

Table 2. Eligibility Criteria

| **Domain** | **Eligibility Criteria** |
| --- | --- |
| **Population** | Adults (≥ 18 years old, include if mean age or majority ≥ 18) |
| **Intervention** | Antimicrobial stewardship programs or strategies focused on improving antimicrobial prescribing (*eg*, clinician education, audit and feedback, public health campaigns, clinical decision support systems, systematic efforts meeting Center for Disease Control and Prevention’s (CDC)[^4^](#_ENREF_4) core elements for outpatient antimicrobial settings, delayed prescribing, *etc*) |
| **Comparator** | Any |
| **Outcomes*** | Reach: type and proportion of patients, community members impacted, type and proportion of providers and facilities  Effectiveness: patient outcomes (*eg*, acceptability, satisfaction, infections successfully treated, adverse events), health care utilization (return clinic visits, emergency room visits, hospital admissions), microbial resistance in the community  Adoption: changes in antimicrobial prescribing, alignment with clinical guidelines  Implementation: costs, staff outcomes (*eg,* acceptability, satisfaction, burn out), other barriers/facilitators  Maintenance: costs, sustainability in prescribing practices (minimum of 12 months) |
| **Timing** | ≥ 6 months |
| **Setting** | Outpatient clinics, emergency rooms, urgent care  Countries that are members of the Organisation for Economic Co-operations and Development (OECD)[^6^](#_ENREF_6) |
| **Study Design** | Randomized controlled trials  Observational studies  Systematic reviews |

Table 3. RE-AIM Framework

| RE-AIM Domain | Review Definition |
| --- | --- |
| Reach | We focused on the type and proportion of patients, community member impacted, in addition to the type and proportion of prescribers, sites and facilities involved |
| Effectiveness | We considered mainly patient outcomes, including resolution of clinical conditions, acceptability or satisfaction with treatments, adverse events, and health care utilization (return clinic visits, ER visits, hospital admissions). We also included changes in microbial resistance in the community as part of the effectiveness of ASPs |
| Adoption | We selected changes in overall antimicrobial prescribing and alignment with clinical guidelines (whether score-based or counts of appropriate/inappropriate prescribing), as these were common measures of the desired changes in clinical practice. |
| Implementation | We included implementation costs, staff outcomes (*eg*, acceptability, satisfaction, burnout), and other barriers/facilitators. |
| Maintenance | We indicated whether studies reported longer-term data on maintenance costs and sustainability of effects (≥ 1 year from start date and presented as a distinct phase from initial implementation). |
